# Evaluation of Privacy Risks of Patients’ Data in China: Case Study

**DOI:** 10.2196/13046

**Published:** 2020-02-05

**Authors:** Mengchun Gong, Shuang Wang, Lezi Wang, Chao Liu, Jianyang Wang, Qiang Guo, Hao Zheng, Kang Xie, Chenghong Wang, Zhouguang Hui

**Affiliations:** 1 Digital China Health Technologies Corporation Limited Beijing China; 2 Shanghai Putuo People's Hospital Tongji University Shanghai China; 3 Department of Radiation Oncology National Cancer Center/National Clinical Research Center for Cancer/Cancer Hospital, Chinese Academy of Medical Sciences and Peking Union Medical College Beijing China; 4 Big Data Center National Cancer Center/National Clinical Research Center for Cancer/Cancer Hospital, Chinese Academy of Medical Sciences and Peking Union Medical College Beijing China; 5 Department of Bioinformatics Hangzhou Nuowei Information Technology Hangzhou China; 6 The Third Research Institute of Ministry of Public Security Key Lab of Information Network Security Ministry of Public Security Shanghai China; 7 Department of VIP Medical Services National Cancer Center/National Clinical Research Center for Cancer/Cancer Hospital, Chinese Academy of Medical Sciences and Peking Union Medical College Beijing China

**Keywords:** patient privacy, privacy risk, Chinese patients’ data, data sharing, re-identification

## Abstract

**Background:**

Patient privacy is a ubiquitous problem around the world. Many existing studies have demonstrated the potential privacy risks associated with sharing of biomedical data. Owing to the increasing need for data sharing and analysis, health care data privacy is drawing more attention. However, to better protect biomedical data privacy, it is essential to assess the privacy risk in the first place.

**Objective:**

In China, there is no clear regulation for health systems to deidentify data. It is also not known whether a mechanism such as the Health Insurance Portability and Accountability Act (HIPAA) safe harbor policy will achieve sufficient protection. This study aimed to conduct a pilot study using patient data from Chinese hospitals to understand and quantify the privacy risks of Chinese patients.

**Methods:**

We used g-distinct analysis to evaluate the reidentification risks with regard to the HIPAA safe harbor approach when applied to Chinese patients’ data. More specifically, we estimated the risks based on the HIPAA safe harbor and limited dataset policies by assuming an attacker has background knowledge of the patient from the public domain.

**Results:**

The experiments were conducted on 0.83 million patients (with data field of *date of birth, gender, and surrogate ZIP codes* generated based on home address) across 33 provincial-level administrative divisions in China. Under the Limited Dataset policy, 19.58% (163,262/833,235) of the population could be uniquely identifiable under the g-distinct metric (ie, 1-distinct). In contrast, the Safe Harbor policy is able to significantly reduce privacy risk, where only 0.072% (601/833,235) of individuals are uniquely identifiable, and the majority of the population is 3000 indistinguishable (ie the population is expected to share common attributes with 3000 or less people).

**Conclusions:**

Through the experiments based on real-world patient data, this work illustrates that the results of g-distinct analysis about Chinese patient privacy risk are similar to those from a previous US study, in which data from different organizations/regions might be vulnerable to different reidentification risks under different policies. This work provides reference to Chinese health care entities for estimating patients’ privacy risk during data sharing, which laid the foundation of privacy risk study about Chinese patients’ data in the future.

## Introduction

### Background

Medical data are naturally distributed across institutions as patients might visit different hospitals at different times or for different diseases. To better understand the risk factors and efficacy of treatment, it is necessary to share data and analyze them. However, patient data are highly sensitive as they contain medical and personal identity information [1-5]. This is a ubiquitous problem. China has the largest population in the world, and the issue of privacy is becoming a big concern for the health care system to share medical data. Inappropriate handling of these sensitive data can lead to privacy leakage, which in turn can result in social embarrassment and commercial fraudulence [6-10].

In the United States, the Health Insurance Portability and Accountability Act (HIPAA) [11] safeguards the health care data. Thus, protected health information can only be considered as deidentified if it is sanitized by one of the following approaches specified by the HIPAA privacy rule [12]: (1) expert determination or (2) safe harbor. The first approach is to recruit an expert with appropriate knowledge and experience to render information with minimal risk to be reidentified. The second approach is to use the safe harbor approach, which explicitly denotes 18 identifiers that need to be removed. The average fine levied for a HIPAA breach is between US $10,000 and US $50,000 per medical record. There are similar guidelines in other countries, for example, the European Union’s General Data Protection Regulation [13] and Canada’s Personal Information Protection and Electronic Documents Act [14], which regulate patient records and other sensitive information. In South Korea (Korea) and Japan, the general law regulating privacy and data protection is the Personal Information Protection Act, and there is a more complete list of international privacy-related laws by country and region.

In China, the *Network Security Law of the People’s Republic of China* [15], which was formally put into effect on June 1, 2017, regulates that network providers must not disclose, falsify, or destroy any personal information they have collected. Any network provider must not disclose this personal information to any third party without obtaining consent from data owners, except for the data that cannot be used to reidentify a specific individual. However, there are no guidelines on how personal information can be processed to satisfy the above regulation. On December 29, 2017, the Chinese government formally released a new regulation called *Information Security Technology and Personal Information Security Specification* (referred to as *Specification*) [16]. In the *Specification*, the Chinese government has clearly defined privacy-related terms such as “personal information controller,” “collection,” “informed consent,” “user portrait,” “personal information security impact assessment,” “deletion,” and “deidentification.” The *Specification* also defines security requirements for different phases (eg, collection, storage, processing, transfer, and disclosure) in handling personal data. However, the *Specification* also has several limitations. First, the *Specification* is a recommended national standard and not a legal regulation; thus, it might not be stringently enforced by different entities. Second, the *Specification* mainly focuses on general purpose information security, where no specific guidance is provided for tackling medical or health care data. For example, in the *Specification*, almost all medical-related data are defined as highly sensitive data. The *Specification*, on one hand, emphasizes the importance of obtaining explicit consent of individuals when collecting, using, or disclosing sensitive personal information, whereas, on the other hand, there are several situations have been added as exceptions. For instance, if the personal information controller is an academic research institution, then it is necessary for them to perform statistical or academic research in public interest. If they provide external academic research or description results with deidentified personal information, they will be exempted from obtaining explicit consent from each individual. In addition, if the use of personal data is directly related to public safety, public health, and major public interest, then there is no need to obtain individual consent on personal data usage. In the third case, if there are certain difficulties in obtaining personal consent and if the use of personal data is to safeguard the major legal rights such as the life and property of the subject or individuals, then such usage of personal information will be exempted from obtaining explicit consent. In the *Specification*, deidentification is defined as a process by which the personal information is technically marked out so that the remaining information cannot be used to reidentify the individual without using additional information. On August 15, 2017, *Information Security Technology and Personal Information Deidentification Specification* was published by the Chinese government for public comments, which also introduced many existing deidentification procedures. However, there is still no clear guidance in China about how to deidentify health care data to ensure sufficient protection of the privacy of individual patients. Owing to the difference in population density, it is also not clear if similar protection mechanisms such as the HIPAA safe harbor rule will provide comparable protection to the Chinese patients’ data. There is also a difference between the external sources of background information that attackers can leverage. For example, there is no public voter’s registry in China, but social networks make public a considerable amount of demographic information of their users such as gender, birthday, school, and job. It is necessary to measure the privacy risks of Chinese patients’ data to better understand the associated privacy risk.

Besides direct identifiers (such as name, national ID, and address), the privacy risk of a medical record is related to the rareness of its key variable values. For example, if there is a unique combination of birthday, gender, and ZIP code, the corresponding record is more likely to be reidentified when compared with records that have duplicated characteristics in the database. It has been shown in the study by Sweeney [17] that 87% of the US population can be uniquely identified by the triplet (birthday, gender, and ZIP code), which reveals a high privacy risk if data are shared without sanitization. It is important to measure the rareness of individual records in a database to understand the potential risk it carries.

Privacy risk measurements and anonymization methods such as *k*-anonymity [18], *l*-diversity [19], *t*-closeness [20], and differential privacy (DP) [21] have motivated many algorithmic and theoretical studies. *k*-anonymity reduces the granularity of data representation using data generalization and suppression technologies. The parameter *k* indicates the number of records within the equivalence class, in which an adversary cannot distinguish an individual. A larger *k* implies a smaller reidentification risk. El Emam et al [22] applied an optimized *k*-anonymity algorithm for health data deidentification. *l*-diversity improves *k*-anonymity by ensuring that the intragroup diversity for sensitive values is controlled by the parameter *l* [19]. *t*-closeness provides a stronger privacy notion than *l*-diversity, where *t*-closeness requires that the distribution of a private attribute in any equivalence class is computationally indistinguishable (ie, no larger than *t*) from the distribution of the attribute in the overall table. Both techniques have been adopted in many medical data deidentification applications [23]. Recently, DP became one of the de facto standards for achieving strong privacy guarantees, which assumes that an attacker with any background knowledge cannot tell if a particular individual’s information has been included or not based on the differentially private outputs [24]. DP technology has also been applied to protect health care data dissemination and analysis [25-27]. In this work, we were interested in a measurement to evaluate the reidentification risks with respect to the HIPAA privacy rule when applied to Chinese patients’ data. However, none of the aforementioned methods can be directly adopted for serving this goal. Therefore, this work resorts to the g-distinct method previously proposed in the study by Malin et al [28] for evaluating reidentification risks of HIPAA-deidentified data in the United States.

### Objectives

The main objectives and contributions of this work are three-fold: (1) to provide one of the first large-scale studies on the privacy risks of Chinese patients’ data, (2) to design specifically experimental studies based on the characteristics of Chinese patients’ data for evaluating the patient privacy risks in China, and (3) to provide references for improving the current privacy protection and rulemaking for Chinese patients’ data.

## Methods

### Data Preprocessing

The datasets used for conducting our experiments are based on cancer patients’ records in the Malignant Cancer Big-data Processing Analysis and Application Research Project (MCBPAARP), which is supported by the National Cancer Institute’s High-Tech Research and Development *863* program. The *863* program was led by the Ministry of Science and Technology of the People’s Republic of China, where the goal of this program is to promote the development of advanced technologies across different fields. This study under MCBPAARP has been approved by the Institutional Review Board of National Cancer Center/National Cancer Hospital and Chinese Academy of Medical Sciences, also known as Ethics Committee of National Cancer Center, National Cancer Hospital, and Chinese Academy of Medical Sciences of Peking Union Medical College under the project ID 2017YFC1311000. In China, hospitals have to update inpatients’ medical record home page to National Health Commission of the People’s Republic of China under a unified standard. The Chinese patients’ data attributes used in this study include fields P5 gender, P6 birthday, and P801 home address (used to generate masked ZIP codes).

**Figure 1:**
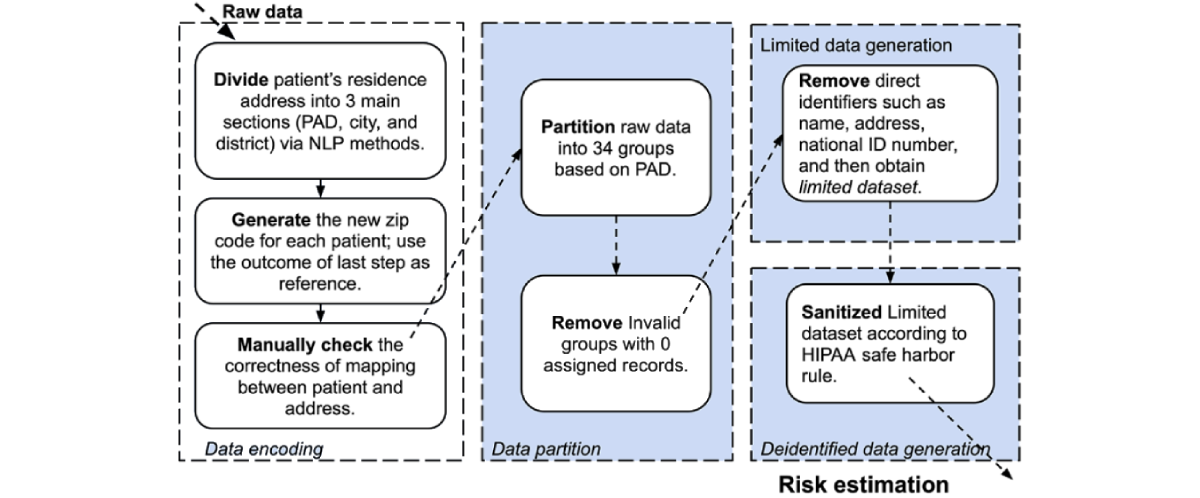
The workflow of raw data preprocessing in this study. HIPAA: Health Insurance Portability and Accountability Act; NLP: neurolinguistic programming; PAD: provincial-level administrative division.

Figure 1 illustrates the methods used for the raw data preprocessing in this study for privacy risk analysis, which includes four phases: (1) data encoding; (2) data partition; (3) limited dataset generation; and (4) *deidentified* dataset generation. The phases are described as follows:

#### Data Encoding

In Chinese patients’ data, the quality of ZIP codes from patients’ raw data is extremely low, which may be either missing or too generalized (ie, only at city level). To overcome this problem, we introduced the following encoding scheme to convert the patient’s address information into geocodes as surrogate ZIP codes in this study. We first divided the patient’s residence address into three sections (ie, provincial-level administrative divisions [PADs], city, and district) by using natural language processing methods. Thereafter, we encoded PAD, city, and district with 2 digits, 3 digits, and 4 digits, respectively, which resulted in surrogate ZIP codes for a total of 9 digits. To ensure the data quality, we conducted two rounds of manual checking for the mapping correctness between the patient’s residence addresses and their surrogate ZIP codes. We excluded patients with missing residence address information and the records with obvious logical error (ie, the patient’s date of birth [DOB] is 1900-01-01). Finally, 0.83 million hospitalized patients’ medical records were selected in this study.

#### Data Partition

We partitioned raw patient data into different groups based on their PADs. Through this phase, we ended up with 33 nonempty PADs except for Hong Kong PAD (see Multimedia Appendix 1 for more details of PADs).

#### Limited Dataset Generation

After the data encoding phase, we further removed additional explicit identifiers, such as name, address, and national ID number from the raw data, which left us with the *limited dataset* with only DOB, gender, and surrogate ZIP codes.

#### Deidentified Dataset Generation

On the basis of the HIPAA safe harbor rule, we further sanitized the limited dataset by generalizing DOB to year and all surrogate ZIP codes to the first 6 digits.

### Risk Evaluation

To evaluate the privacy risk of the preprocessed Chinese patients’ data, we adopted the g-distinct method introduced in the study by Malin et al [28] for studying a similar problem in the United States. The g-distinct method quantifies the uniqueness of individual records within a database, where an individual is said to be unique if such an individual has a combination of personal attributes that no other individuals in the same dataset has. Furthermore, we say an individual is g-distinct if the combination of their attributes is identical to at the most g-1 other individuals in the whole dataset space. For example, suppose an individual has the following combination of attributes: age at 35 years and gender as male. If there does not exist any other individual whose age and gender are also 35 years and male, respectively, then such an individual is considered as unique (ie, 1-distinct). In addition, if the total number of individuals with the same combination of attributes is equal to *k,* then we state this individual is *k-distinct.*

In other words, we can also describe the g-distinct as the sum over the number of patients in all bins with less than or equal to *g* individuals, which can be written as shown in equation (1):

h(g)=\sum_{i=1}^{g}i|bin(i)| (1)

Here *g* denotes the parameter and |bin(i)| refers to the number of bins with exact *i* patients having identical attributes. This measurement serves as a proxy to the risk of stratified population with different combinations of characteristics. In this study, we applied the above *g*-distinct metric to the Chinese patients’ data to evaluate the privacy risk.

## Results

### Experimental Setup

The g-distinct analysis is a population inspection method that allows us to investigate a particular cross-section for specific population collection. Such particular cross-section represents the set of individuals whose private records are most vulnerable to reidentification attacks. In our experiments, we conducted g-distinct analysis over the limited dataset (ie, DOB, gender, and ZIP code) and the safe harbor dataset (ie, birth year, gender, and masked ZIP code) to examine how the safe harbor data can improve the privacy of individual patients over limited data.

### Experimental Results

**Figure 2:**
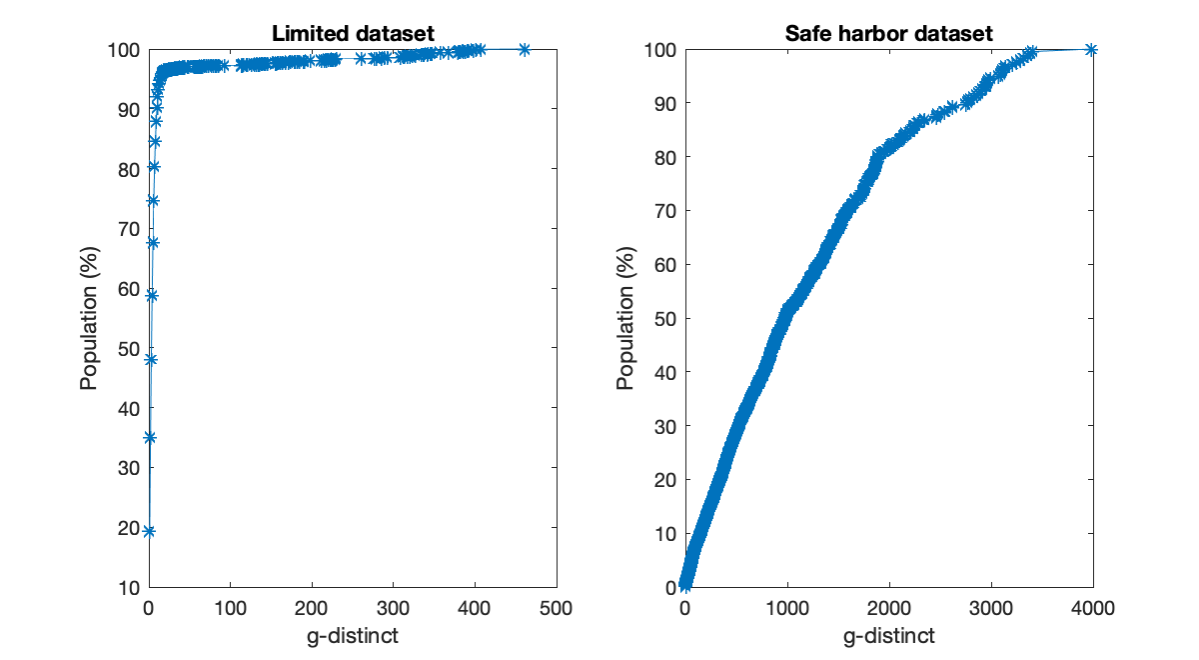
The g-distinct versus percentage of population under limited dataset and safe harbor dataset, respectively.

The results of g-distinct analysis based on nationwide datasets for both the safe harbor dataset and limited dataset are illustrated in Figure 2. In Figure 2, the left and right subgraphs represent the g-distinct analysis results for limited and safe harbor datasets, respectively. According to the nationwide g-distinct analysis results, we have two major observations. On the one hand, without sufficient deidentification process (ie, the limited data on the left), the whole dataset is highly risky. For instance, 19.58% (163,262/833,235) of the population is 1-distinct in the limited dataset (ie, uniquely identifiable under the g-distinct metric). In addition, more than 90.6% of the population is 10-distinct, which implies that the majority of the population in the limited dataset is expected to share common attributes with 10 or less people. Such sheer number of distinct individuals results in a huge difficulty for privacy protection. Thus, in such cases, the limited data are extremely vulnerable to reidentification attacks. On the other hand, as shown in Figure 2, the safe harbor dataset is able to significantly preserve the patient’s privacy, in which only 0.072% (601/833,235) of individuals are uniquely identifiable (ie, 1-distinct), and the majority of the population (around 95%) is 3000 indistinguishable.

**Figure 3:**
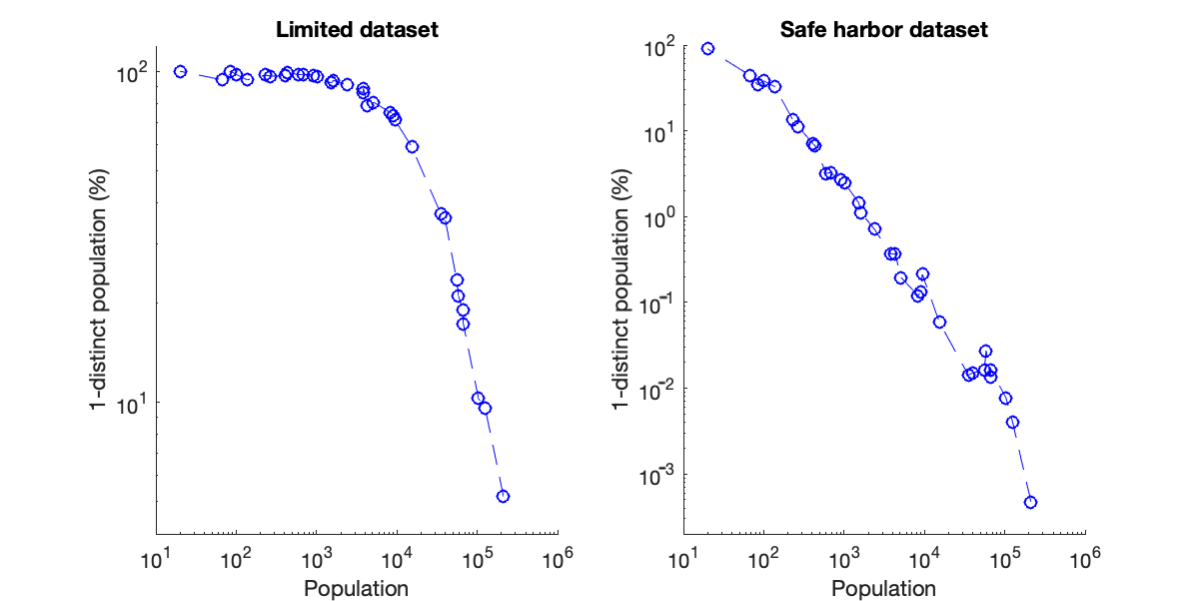
Percentage of 1-distinct versus total population under limited dataset and safe harbor dataset, respectively.

We also studied the relationship between distinct individuals and the underlying populations, which simulates the impact of different ZIP code–masking strategies on the privacy protection. The results of this experiment have been illustrated in Figure 3. There are a total of 34 PADs in China. As there were no patient records with residence address within Hong Kong in the collected datasets, we estimated the percentage of 1-distinct population over the other 33 PADs (see Multimedia Appendix 2 for more details). Figure 3 shows the percentage of 1-distinct population associated with each PAD in an ascending order of the sample population in the given PAD. The 2 subgraphs are the results over limited dataset and the safe harbor dataset, respectively. Owing to the accommodation of different scales of 1-distinct percentage along with the increase in population, the 2 plots are depicted in log-log scale. As shown in Figure 3, both results show a similar tendency, where the percentage of 1-distinct population decreases as the sampled population increases in different PADs. This is because a PAD with more sampled population tends to result in a higher probability to have more than 1 patient who shares the same attributes. Another observation from the result is that when the sampled population increases, the percentage of 1-distinct population of the safe harbor dataset decreases dramatically. When the population has increased to 10,000, the 1-distinct percentage has already dropped to 0.05%. In contrast, the decreasing tendency for the limited dataset seems more moderate (ie, potentially higher privacy risks). We can see that there is still 5% population more with 1-distinct for a PAD of 200,000 population in the limited dataset.

## Discussion

### Principal Findings

This work is one of the first large-scale studies on evaluating privacy risks of Chinese patients’ data, which analyzed the reidentification risks based on the HIPAA safe harbor and limited dataset policies. The originality of this work can be summarized in three ways:

1. Originality in exploring new observations: Although many Chinese Acts [15] and national specification/regulations [16] have cited the HIPAA safe harbor rule [11] as a reference standard for guiding patient data deidentification in China, there is still a lack of quantitative observation on the reidentification risk of Chinese patient data when applying the HIPAA safe harbor standard. This work provides one of the first quantitative studies on large-scale nationwide Chinese patients’ data toward this goal.
2. Originality in designing new experiments: Some Chinese patients’ data attributes are unique and different from those in the United States. Therefore, these data cannot be applied directly to the risk assessment method used in previous US studies. For example, Chinese patients’ data typically have extremely low quality of ZIP codes, which may be either missing or too generalized (ie, only at city level). Thus, we designed new data encoding, data partition, and data masking schemes based on Chinese patients’ data characteristics to meet this goal.
3. Originality in contributing new knowledge: We made an assumption that the risk evaluation scheme defined by the HIPAA is satisfactory with respect to Chinese patient data as well. According to this assumption, we designed and implemented our experimental studies based on Chinese patients’ data. As patient privacy protection is a very important topic, many other research studies have been conducted in Europe [29-31], Japan [32,33], and Australia [34]. However, most of these studies are more qualitative in orientation and usually not suitable for Chinese patients’ data, which mainly focus on the interpretation and comparison of laws and regulations. In contrast, the focus of this work was to quantify the Chinese patient privacy risks with large-scale and real-world patient data collected from China. According to our experimental studies, our assumption is supported by the results of this work, which illustrates findings similar to those of a previous US study by Malin et al [28] that evaluated reidentification of US patient data associated with the HIPAA policies. Such studies are amenable to various kinds of meta-evaluations, enabling administrative roles such as government’s policy makers and datacenter administrators to be able to evaluate policies and to determine the potential impact on reidentification risk. The experimental results demonstrate the power of the g-distinct analysis applied on Chinese patients’ data. In general, according to the experimental results, the safe harbor dataset provides much stronger privacy protection in terms of 1-distinct than that provided by the limited dataset in Chinese patients’ data.

### Limitations

In general, this work provides justification for reidentification risk estimates on Chinese patient records before sharing data. However, the proposed studies still have a few limitations. First, the privacy risk that we estimated for the case study is based on the cancer patients’ data without including patients with other diseases. Second, although the datasets are from 33 of 34 PADs, the study is still limited by the data scale, which only covers less than 0.06% of the Chinese population (ie, 0.83 million patients’ records vs 1.5 billion total population). Third, the demographic information used in this study is also limited. For example, it is unfeasible to measure the identifiability based on nationality. Therefore, raw data are collected with selection bias because of aforementioned limitations. All these limitations justify further investigation along this line.

### Conclusions

The study of Chinese patients’ privacy risk in this work fills the gap of the privacy research between the United States and China. Moreover, as the Chinese government has not yet issued specific regulations or policies directly against privacy protection of citizens’ health data, our experimental studies have the potential for Chinese officials to improve current health data–sharing regulations. The policy might vary largely among provinces, as according to the statistics, the g-distinct measurements vary widely across the provinces as well. Privacy officials might issue flexible policies for different regions.

## Appendix

Multimedia Appendix 1 Abbreviations of provinces in China.

Multimedia Appendix 2 The g-distinct analysis results.

## Abbreviations

DOB: date of birth
DP: differential privacy
HIPAA: Health Insurance Portability and Accountability Act
MCBPAARP: Malignant Cancer Big-data Processing Analysis and Application Research Project
PAD: provincial-level administrative division
