# Supplementary material for: Evaluation of Privacy Risks of Patients’ Data in China: Case Study
Source: JMIR Med Inform. 2020 Feb 5;8(2):e13046. doi: 10.2196/13046 (PMC7055805; doi:10.2196/13046)
Supplement: Multimedia Appendix 1 [file medinform_v8i2e13046_app1.docx]

**Multimedia Appendix 1**. Abbreviations of provinces in China

All abbreviations of provinces in China uses the GB/T 2260-2007 [[35]](https://paperpile.com/c/8Jcluj/Nck6) standard, the details are listed as follows:

| **AH** | Anhui | **BJ** | Beijing | **FJ** | Fujian |
| --- | --- | --- | --- | --- | --- |
| **GS** | Gansu | **GD** | Guangdong | **GX** | Guangxi |
| **GZ** | Guizhou | **HI** | Hainan | **HE** | Hebei |
| **HA** | Henan | **HL** | Heilongjiang | **HB** | Hebei |
| **HN** | Hunan | **JL** | Jilin | **JS** | Jiangsu |
| **JX** | Jiangxi | **LN** | Liaoning | **NM** | Inner Mongolia |
| **NX** | Ningxia | **QH** | Qinghai | **SD** | Shandong |
| **SX** | Shanxi | **SN** | Shaanxi Province | **SH** | Shanghai |
| **SC** | Sichuan | **TJ** | Tianjin | **XZ** | Tibet |
| **XJ** | Xinjiang | **YN** | Yunnan | **ZJ** | Zhejiang |
| **CQ** | Chongqing | **TW** | Taiwan | **HK** | Hongkong |
| **MO** | Macau |  |  |  |  |
