# Supplementary material for: Evaluation of Privacy Risks of Patients’ Data in China: Case Study
Source: JMIR Med Inform. 2020 Feb 5;8(2):e13046. doi: 10.2196/13046 (PMC7055805; doi:10.2196/13046)
Supplement: Multimedia Appendix 2 [file medinform_v8i2e13046_app2.docx]

**Multimedia Appendix 2**. g-distinct Analysis Result

| **PAD** | **Patient Population** | **# of 1-distinct patient**  **(Limited)** | **% of 1-distinct patient**  **(Limited)** | **# of 1-distinct patient**  **(Safe Harbor)** | **% of 1-distinct patient**  **(Safe Harbor)** |
| --- | --- | --- | --- | --- | --- |
| AH | 3817 | 3283 | 86.00995546 | 14 | 0.366780194 |
| BJ | 8359 | 6270 | 75.00897237 | 10 | 0.119631535 |
| FJ | 677 | 663 | 97.93205318 | 22 | 3.249630724 |
| GS | 35244 | 13052 | 37.03325389 | 5 | 0.014186812 |
| GD | 595 | 581 | 97.64705882 | 19 | 3.193277311 |
| GX | 228 | 222 | 97.36842105 | 31 | 13.59649123 |
| GZ | 4329 | 3411 | 78.79417879 | 16 | 0.36960037 |
| HI | 85 | 85 | 100 | 29 | 34.11764706 |
| HE | 212396 | 11035 | 5.195483907 | 1 | 0.000470819 |
| HA | 125551 | 12042 | 9.591321455 | 5 | 0.003982445 |
| HL | 103677 | 10636 | 10.25878449 | 8 | 0.007716273 |
| HB | 1532 | 1412 | 92.16710183 | 22 | 1.436031332 |
| HN | 67053 | 11585 | 17.2773776 | 11 | 0.016404933 |
| JL | 2388 | 2174 | 91.03852596 | 17 | 0.711892797 |
| JS | 15381 | 9071 | 58.97535921 | 9 | 0.058513751 |
| JX | 1016 | 976 | 96.06299213 | 25 | 2.460629921 |
| LN | 58649 | 12273 | 20.926188 | 16 | 0.027280943 |
| NM | 9416 | 6720 | 71.36788445 | 20 | 0.212404418 |
| NX | 268 | 260 | 97.01492537 | 30 | 11.19402985 |
| QH | 407 | 394 | 96.80589681 | 29 | 7.125307125 |
| SD | 3840 | 3403 | 88.61979167 | 14 | 0.364583333 |
| SX | 5134 | 4137 | 80.5804441 | 10 | 0.194779899 |
| SN | 904 | 880 | 97.34513274 | 24 | 2.654867257 |
| SH | 99 | 97 | 97.97979798 | 38 | 38.38383838 |
| SC | 8998 | 6627 | 73.64969993 | 12 | 0.13336297 |
| TJ | 438 | 434 | 99.08675799 | 29 | 6.621004566 |
| XZ | 68 | 64 | 94.11764706 | 30 | 44.11764706 |
| XJ | 55563 | 12964 | 23.3320735 | 9 | 0.016197829 |
| YN | 66032 | 12554 | 19.01199418 | 9 | 0.013629755 |
| ZJ | 1618 | 1519 | 93.88133498 | 18 | 1.112484549 |
| CQ | 39724 | 14289 | 35.97069781 | 6 | 0.015104219 |
| TW | 137 | 129 | 94.16058394 | 45 | 32.84671533 |
| HK | 0 | 0 | 0 | 0 | 0 |
| MO | 20 | 20 | 100 | 18 | 90 |
